# Evaluation of ChatGPT-5 for Automated ASPECTS Assessment on Non-Contrast CT in Acute Ischemic Stroke

**DOI:** 10.3390/diagnostics15243160

**Published:** 2025-12-11

**Authors:** Samet Genez, Hamza Özer, Ayşenur Buz Yaşar, Yunus Yılmazsoy, Tunahan Soydan, Abdullah Emre Sarıoğlu, Sadettin Ersoy

**Affiliations:** 1 Department of Radiology, Faculty of Medicine, Bolu Abant Izzet Baysal University, Bolu 14030, Turkey; 2 Department of Neurology, Faculty of Medicine, Bolu Abant Izzet Baysal University, Bolu 14030, Turkey

**Keywords:** acute ischemic stroke, ASPECTS, ChatGPT-5, non-contrast CT, artificial intelligence

## Abstract

**Background/Objectives**: This study aimed to evaluate the ability of ChatGPT-5, a multimodal large language model, to perform automated ASPECTS assessment on non-contrast CT (NCCT) in patients with acute ischemic stroke. **Methods**: This retrospective, single-center study included 199 patients with anterior circulation AIS who underwent baseline NCCT before reperfusion therapy between November 2020 and February 2025. Each NCCT was evaluated by two human readers and by ChatGPT-5 using four representative images (two ganglionic and two supraganglionic). Interobserver agreement was measured with the intraclass correlation coefficient (ICC), and prognostic performance was analyzed using multivariable logistic regression and receiver operating characteristic (ROC) analysis for 3-month functional independence (mRS ≤ 2). **Results**: ChatGPT-5 demonstrated good-to-excellent agreement with expert consensus (ICC = 0.845; 95% CI, 0.792–0.884; κ = 0.79). ChatGPT-ASPECTS were independently associated with 3-month functional independence (OR = 1.28 per point; *p* = 0.004), comparable to consensus-ASPECTS (OR = 1.31; *p* = 0.003). Prognostic discrimination was similar between ChatGPT-5 and consensus scoring (AUC = 0.78 vs. 0.80; *p* = 0.41). **Conclusions**: ChatGPT-5 achieved high reliability and strong prognostic validity in automated ASPECTS assessment without task-specific training. These findings highlight the emerging potential of large language models for quantitative image interpretation, though clinical implementation will require multicenter validation and regulatory approval.

## 1. Introduction

Acute ischemic stroke (AIS) remains one of the leading causes of mortality and long-term disability worldwide [1]. Rapid and accurate evaluation of early ischemic changes (EIC) on initial non-contrast computed tomography (NCCT) is essential for clinical decision making, particularly in selecting candidates for reperfusion therapies such as intravenous thrombolysis and mechanical thrombectomy [2,3].

The Alberta Stroke Program Early CT Score (ASPECTS) provides a validated and systematic 10-point scale for quantifying EIC within the middle cerebral artery (MCA) territory and serves as an integral component of modern stroke triage protocols [4].

Despite its clinical utility, manual ASPECTS assessment is subjective and prone to interobserver variability, leading to inconsistencies that may affect patient selection and outcome prediction [5,6]. This challenge has prompted the development of artificial intelligence (AI) tools, especially those based on deep learning (DL) and machine learning to automate ASPECTS assessment and enhance reproducibility [7,8]. In previous studies, dedicated automated ASPECTS software and DL-based models have demonstrated non-inferior performance to human experts, achieving good-to-excellent agreement while markedly reducing the time required for image interpretation and triage decisions [2,9].

The emergence of large language models (LLMs) represents a new frontier for artificial intelligence in medical imaging [10]. Trained on vast multimodal datasets, these models are capable of processing both textual and visual information, enabling applications beyond traditional natural language processing tasks [11,12]. Recent iterations such as GPT-4 and GPT-4o have shown substantial improvements in logical reasoning and accuracy on medical image-based examinations [13,14]. However, their reliability in performing highly specialized quantitative image interpretation tasks, such as ASPECTS assessment, has not yet been systematically validated.

This study aimed to evaluate the ability of ChatGPT-5 for automated ASPECTS assessment on NCCT scans in patients with AIS to support rapid and objective decision making in stroke triage.

## 2. Materials and Methods

### 2.1. Study Design and Patient Selection

This retrospective, single-center study was conducted at the Department of Radiology, Bolu Abant Izzet Baysal University Faculty of Medicine, and approved by the institutional ethics committee (approval number: 2025/415). Due to the retrospective nature of the study, the requirement for written informed consent was waived. Patients who presented with AIS between November 2020 and February 2025 and underwent baseline NCCT before reperfusion therapy were included. Inclusion criteria were as follows: (1) acute anterior circulation infarction involving the MCA territory, (2) availability of diagnostic-quality NCCT images, and (3) complete clinical, angiographic, and outcome data. Patients with posterior circulation infarctions, significant motion artifacts, chronic parenchymal sequelae, hemorrhagic transformation, or incomplete data were excluded to restrict the cohort to anterior circulation strokes, for which ASPECTS is validated, and to minimize image-quality and chronic lesion confounders that could compromise reliable ASPECTS assessment.

### 2.2. Imaging Protocol

All NCCT examinations were performed on a 64-slice multidetector CT scanner (Revolution EVO, GE Healthcare, Milwaukee, WI, USA) using a standardized protocol: 120 kV, 320 mAs, 0.5 mm detector collimation, gantry rotation 400 ms, pitch 0.641, FOV 450–500 mm, and axial slice thickness 5 mm (with thin-section reconstructions available as needed).

### 2.3. ASPECTS Assessment by Human Readers

Each baseline NCCT was independently evaluated by one radiologist (≥5 years of experience in general radiology) and one neurologist (≥5 years of experience in stroke imaging) using the ASPECTS method [4]. ASPECTS was determined on two standard axial NCCT levels: the ganglionic level (through the thalamus and basal ganglia) and the supraganglionic level (at the level of the corona radiata). The MCA territory was divided into 10 regions: the caudate, lentiform nucleus, internal capsule, insula, and six cortical regions (M1–M6). One point was subtracted for each region showing early ischemic change, defined as parenchymal hypoattenuation or focal loss of gray–white matter differentiation relative to the contralateral hemisphere. Final ASPECTS values ranged from 0 to 10.

Both readers were blinded to all clinical and angiographic information and to each other’s results. In cases of disagreement, a consensus score was established by a neuroradiologist with more than 10 years of experience, whose decision was accepted as the reference standard (ASPECTS-consensus).

### 2.4. AI-Based ASPECTS Assessment Using ChatGPT-5

For the automated assessment, ASPECTS scoring was performed using ChatGPT-5 (OpenAI, San Francisco, CA, USA), accessed through the web-based ChatGPT interface (https://chatgpt.com) with the vision-capable model and default settings, without any fine-tuning or Custom GPT. For each patient, four representative axial NCCT images (two at the ganglionic level and two at the supraganglionic level corresponding to standard ASPECTS planes) were exported from the PACS as 8-bit grayscale JPEG files (512 × 512 pixels) [2,9]. These images were uploaded into ChatGPT-5, and the following standardized prompt was entered for each case: “Please determine the ASPECT score from these non-contrast head CT images for the right/left hemisphere affected by acute ischemic stroke.” Each patient was analyzed in a separate chat session to prevent model carryover effects from previous evaluations [15]. Each case was analyzed once using the hosted ChatGPT-5 model via the web-based interface. File names were anonymized, and any potentially identifiable personal information embedded in the image metadata was removed to ensure complete data anonymization and patient confidentiality [16].

### 2.5. Clinical and Angiographic Data

Clinical and demographic variables, including age, sex, vascular risk factors (hypertension, diabetes mellitus, coronary artery disease, atrial fibrillation, and hyperlipidemia), and stroke laterality, were collected from the electronic medical record system. Angiographic outcomes were evaluated using the modified Thrombolysis in Cerebral Infarction (mTICI) scale; successful reperfusion was defined as TICI 2b–3. Functional outcome was determined using the modified Rankin Scale (mRS) at 3 months post-stroke; mRS ≤ 2 was defined as functional independence [17]. Hemorrhagic transformation (HT) was evaluated on follow-up imaging within 48 h after treatment and classified according to the Heidelberg Bleeding Classification [18].

### 2.6. Statistical Analysis

All statistical analyses were performed using R software (version 4.3.1, R Foundation for Statistical Computing, Vienna, Austria). The distribution of continuous variables was assessed using the Shapiro–Wilk test. Continuous data were summarized as mean ± standard deviation when approximately normally distributed and as median (interquartile range) otherwise. For group comparisons of continuous variables, Student’s *t*-test was used for normally distributed variables and the Mann–Whitney U test for non-normally distributed variables. Categorical variables were compared using the Chi-square test or Fisher’s exact test, as appropriate. These tests were chosen according to the measurement scale and distribution of the variables and are standard methods in stroke outcome research. To evaluate the interobserver agreement among the three assessors (radiologist, neurologist, and ChatGPT-5), the Intraclass Correlation Coefficient (ICC) was calculated. ICC analysis was performed using a two-way random effects model under the assumption of absolute agreement. ICC values were interpreted as follows: below 0.50, poor; between 0.50 and 0.75, moderate; between 0.75 and 0.90, good; and above 0.90, excellent agreement. Agreement between ChatGPT-5 and consensus was further assessed with Bland–Altman analysis. For functional independence (3-month mRS ≤ 2), multivariable logistic regression models included ASPECTS (per 1-point increase) and clinical covariates (age, sex, successful reperfusion [mTICI 2b–3], and other prespecified factors where applicable). Model discrimination was assessed using the area under the receiver operating characteristic (ROC) curve (AUC), with pairwise comparisons performed using the DeLong test. Statistical significance was set at *p* < 0.05.

## 3. Results

A total of 199 patients (101 men and 98 women; mean age, 70.2 ± 12.5 years) with acute AIS were included between November 2020 and February 2025. Baseline clinical and angiographic characteristics, including vascular risk factors, occlusion sites, and treatment details, are summarized in Table 1.

Pairwise interobserver reliability among ChatGPT-5, the radiologist, and the neurologist demonstrated good-to-excellent agreement (ICC range, 0.807–0.854; all *p* < 0.001) (Table 2). Specifically, ICC values were 0.854 (95% CI, 0.797–0.894) between the radiologist and neurologist, 0.845 (95% CI, 0.792–0.884) between ChatGPT-5 and the consensus, 0.821 (95% CI, 0.765–0.868) between ChatGPT-5 and the radiologist, and 0.807 (95% CI, 0.745–0.856) between ChatGPT-5 and the neurologist. Overall, three-rater agreement was low (ICC [2,1] = 0.451; 95% CI, 0.335–0.555; *p* < 0.001), reflecting variability among all raters. For dichotomized ASPECT categories (<7 vs. ≥7), ChatGPT-5 and consensus readers showed substantial categorical agreement (Cohen’s κ = 0.79; 95% CI, 0.71–0.86; *p* < 0.001).

**Figure 1:**
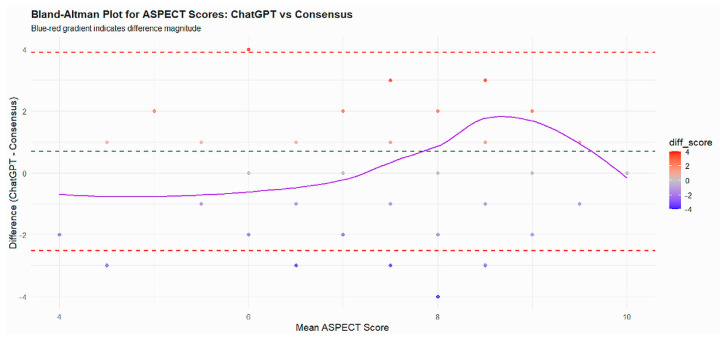
The Bland–Altman plot illustrates the agreement between ASPECT scores obtained from ChatGPT and the consensus scoring by experts. Each point represents a patient, plotted according to the mean of the two scores (x-axis) and the difference between ChatGPT and consensus scores (y-axis). The solid purple line represents the LOESS trend, showing how the differences vary with the mean ASPECT score. The green dashed line indicates the mean difference (bias), and the red dashed lines represent the limits of agreement (±1.96 SD). Most differences cluster around zero, and the majority fall within the limits of agreement, indicating good agreement between ChatGPT and consensus scores. Slight deviations at the extremes suggest minor over- or underestimation by ChatGPT in very low or high ASPECT scores, but overall reliability is high.

The mean ASPECT score assigned by ChatGPT-5 (8.64 ± 1.78) was slightly higher than that of the consensus (7.96 ± 1.36), yielding a mean difference of 0.68 (95% CI, 0.42–0.93; *p* < 0.001). Both ASPECTS distributions fulfilled the normality assumption on Shapiro–Wilk testing; therefore, the within-patient difference between ChatGPT-5 and consensus scores was evaluated using a paired *t*-test. The Bland–Altman plot demonstrated a small positive bias, with ChatGPT-5 assigning on average slightly higher ASPECTS values than the neuroradiology consensus. Most data points clustered near the zero-difference line and within the 95% limits of agreement, indicating good concordance overall, whereas larger positive differences were mainly observed at higher mean ASPECTS values, consistent with a mild tendency of ChatGPT-5 to overestimate ASPECTS in cases with relatively limited ischemic changes (Figure 1).

**Figure 2:**
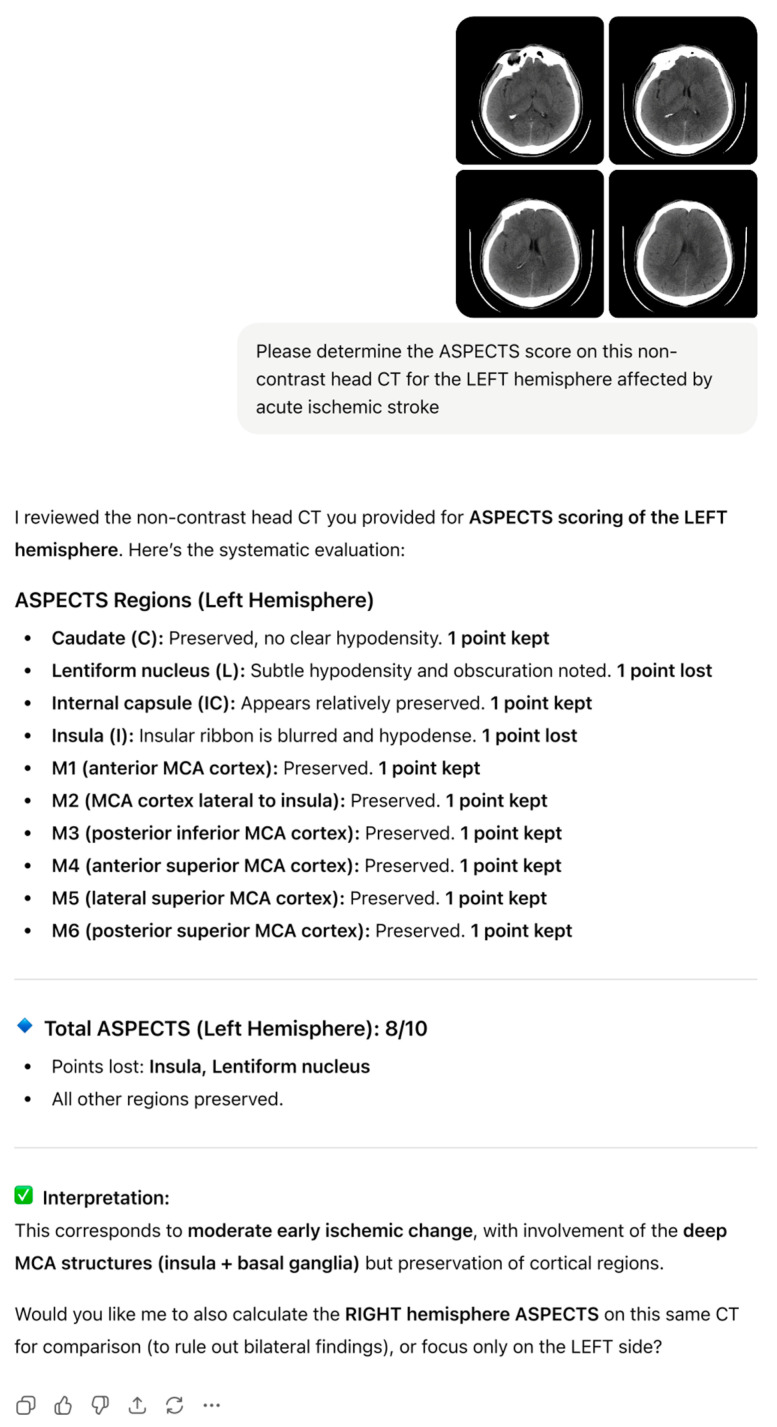
ChatGPT-5-generated ASPECTS assessment for left MCA ischemia on baseline NCCT, with region-wise scoring and total ASPECTS.

**Figure 3:**
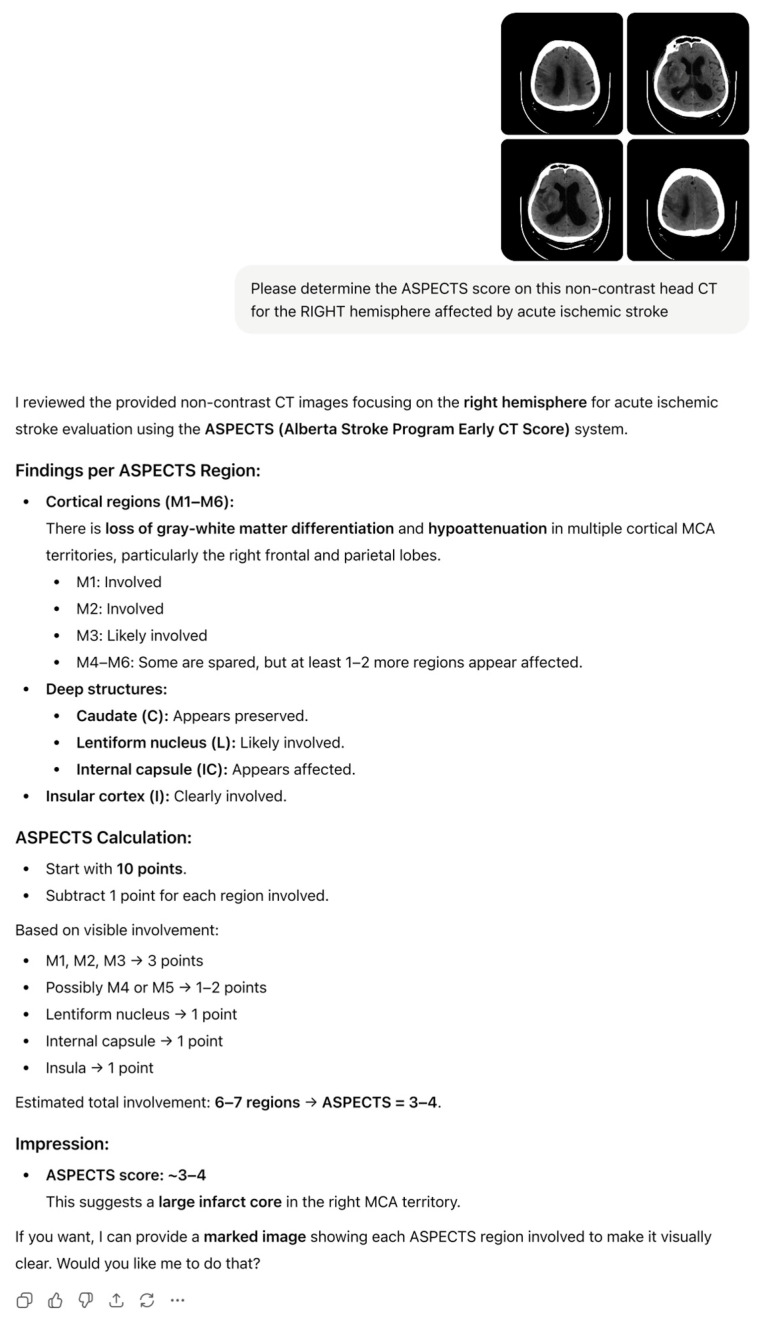
ChatGPT-5-generated ASPECTS assessment for right MCA ischemia with a large infarct core, showing regional involvement and estimated total ASPECTS.

For both ChatGPT-5 and consensus ASPECT scores dichotomized as <7 or ≥7, chi-square analyses showed no significant associations with mTICI success, functional independence, or HT (all *p* > 0.25). Representative examples of ChatGPT-5 output, including region-wise ASPECTS breakdown and narrative interpretation for the left and right hemispheres, are shown in Figure 2 and Figure 3.

**Figure 4:**
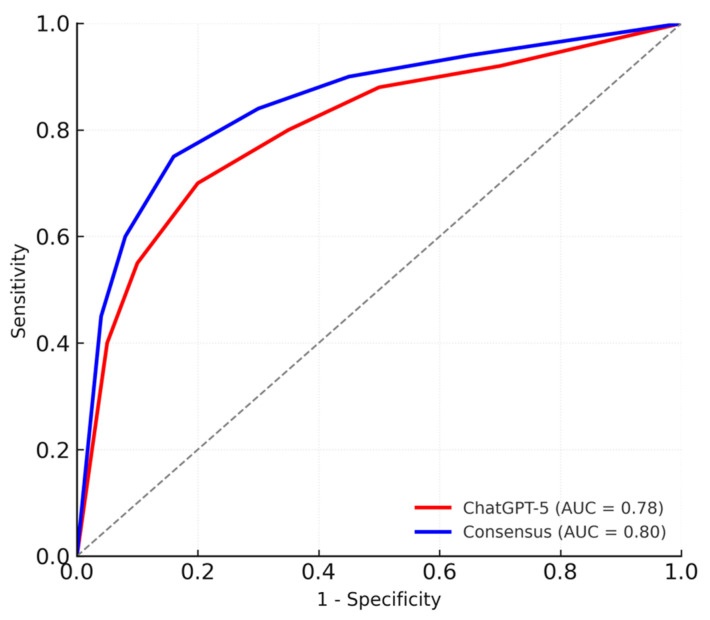
ROC Curves Comparing ChatGPT-5 and Consensus ASPECTS for Predicting Functional Independence.

In multivariable logistic regression, higher ChatGPT-ASPECTS were independently associated with 3-month functional independence (mRS ≤ 2) (OR = 1.28 per point; 95% CI: 1.09–1.52; *p* = 0.004). When the same model was applied using consensus-ASPECTS, comparable associations were observed (OR = 1.31 per point; 95% CI: 1.11–1.54; *p* = 0.003) (Table 3). In both models, ASPECTS was entered as a continuous predictor (per 1-point increase), which allowed us to use the full information of the scale rather than relying solely on dichotomized categories. The discriminative ability of the logistic regression models was also comparable between ChatGPT-5 and consensus scoring (AUC = 0.78 vs. 0.80; *p* = 0.41, DeLong test), with both ROC curves lying well above the diagonal reference line and closely overlapping across the full range of 1 − specificity, indicating similar and good discrimination for 3-month functional independence (Figure 4).

## 4. Discussion

This study evaluated the ability of ChatGPT-5 to perform automated ASPECTS assessment on NCCT scans in patients with AIS. Using a standardized prompt and four representative NCCT images, ChatGPT-5 demonstrated excellent reliability and substantial concordance with expert consensus, suggesting potential utility as an assistive tool for rapid stroke triage.

The interobserver reliability analysis revealed strong agreement between ChatGPT-5 and the expert consensus (ICC = 0.845; 95% CI, 0.792–0.884), meeting the threshold for good-to-excellent agreement. This level of consistency is comparable to that reported for several dedicated DL systems developed for automated ASPECTS assessment, such as the Heuron ASPECTS software (ICC = 0.78) and other convolutional network-based models (mean ICC ≈ 0.72–0.83) [7,19,20]. These findings demonstrate that a general-purpose multimodal LLM, when guided by task-specific prompts and targeted image selection, can achieve diagnostic reliability previously attainable only with specially designed DL architectures [13,21,22]. Moreover, categorical agreement at the clinically relevant dichotomized threshold (ASPECTS < 7 vs. ≥7; κ = 0.79) further supports ChatGPT-5’s consistency for binary clinical decision making.

Previous studies on automated ASPECTS assessment have mainly used deep learning models trained on large stroke imaging datasets. These systems have shown high accuracy, with reported sensitivities around 90% and overall accuracies close to 83% [9,19,21]. Despite their success, they require extensive dataset preparation and model training, and their performance can vary when applied to scans from different institutions or imaging protocols [23,24,25]. In contrast, ChatGPT-5 is a general-purpose AI model that is not specifically trained on stroke imaging. Its comparable reliability demonstrates that LLMs can perform structured quantitative interpretation through cross-modal reasoning without task-specific retraining [26].

Recent studies have shown that LLMs such as GPT-4 can perform advanced reasoning in radiology reporting, diagnostic decision support, and quality improvement tasks [27,28]. These text-based applications demonstrate their adaptability to radiologic workflows and clinical reasoning.

Previous evaluations of multimodal LLMs in imaging domains such as musculoskeletal, chest, and abdominal radiography have typically shown moderate diagnostic accuracy, suggesting that model performance depends heavily on the task structure [22,29]. The high reliability observed in ASPECTS assessment may be related to its reliance on symmetry-based lesion detection and binary regional assessment, which appear to align well with the visual reasoning patterns observed in LLMs [21].

Pairwise ICC values between ChatGPT-5 and each human reader, and between the two human readers, were good-to-excellent, indicating high overall agreement; however, the overall three-rater ICC was low, likely reflecting systematic level shifts in scoring behavior rather than random unreliability of the model. In line with this, ChatGPT-5 tended to assign slightly higher ASPECT scores than human experts (mean difference = 0.68, *p* < 0.001), indicating a mild conservative bias that may lead to underestimation of ischemic extent. This conservative tendency seemed to be most pronounced in cases with very subtle early ischemic changes (near-threshold loss of gray–white differentiation or minimal insular ribbon blurring), in which neuroradiology experts judged regional involvement while ChatGPT-5 frequently retained a higher ASPECTS value. Such borderline findings are a well-known source of interobserver variability and likely account for a substantial proportion of the outlier cases with larger disagreement between human and AI-based ASPECTS assessment. Similar upward scoring trends have been reported with previous DL systems, likely reflecting the model’s tendency to avoid false-positive lesion detection in mildly hypo-attenuated regions [7]. Despite this minor difference, ChatGPT-ASPECTS were independently associated with 3-month functional independence (mRS ≤ 2) in multivariable analysis (OR = 1.28 per point, *p* = 0.004), consistent with the consensus results (OR = 1.31, *p* = 0.003). Both ChatGPT-5 and consensus scoring achieved comparable prognostic performance for functional outcomes (AUC = 0.78 vs. 0.80, *p* = 0.41, DeLong test), suggesting that ChatGPT-5 can extract clinically meaningful imaging information rather than relying solely on pixel-level pattern recognition.

No significant relationship was observed between ASPECTS categories and either mTICI success or HT, consistent with previous studies showing that ASPECTS is primarily predictive of functional outcomes [30,31].

Given its diagnostic reliability, ChatGPT-5’s near-human accuracy suggests potential as a supplementary decision-support tool, particularly in centers lacking dedicated neuroradiology expertise, as it can directly interpret standard DICOM-converted images and generate an ASPECTS estimate within seconds of NCCT acquisition. This accessibility and speed may enhance the applicability of AI-assisted triage, especially in community hospitals and resource-limited environments. At the same time, any automated ASPECTS tool carries a risk of diagnostic error, including under- or overestimation of ischemic changes, and therefore must be used under careful human supervision. Nevertheless, real-world implementation will require addressing issues of reproducibility and regulatory compliance. Therefore, ChatGPT-5 should presently be regarded as a research-grade adjunct rather than an independent clinical decision engine [28,29,32].

An additional conceptual limitation is that ChatGPT-5 is a general-purpose LLM rather than a model specifically optimized for clinical or radiology tasks. In biomedical applications, further performance gains often require domain-adaptation strategies such as retrieval-augmented generation (RAG), fine-tuning on curated medical datasets, and systematic prompt engineering with human feedback. These approaches can help reduce hallucinations, improve alignment with expert knowledge, and increase task fidelity, but they also demand high-quality labeled data, rigorous validation, and additional computational infrastructure. Exploring such strategies for LLM-based ASPECTS assessment and acute stroke imaging represents a natural direction for future work [33].

This study has several limitations. First, its retrospective, single-center design limits the generalizability of the findings. Differences in CT scanner types, acquisition parameters, and reconstruction protocols across institutions may influence model performance. Second, we used a single run of the hosted ChatGPT-5 model for each case and did not systematically repeat the analyses to quantify intra-model variability. Moreover, because ChatGPT-5 is a closed-source, cloud-based system that can be updated by the vendor, the exact internal model state at the time of our experiment cannot be fully reproduced; future studies should incorporate explicit model versioning and repeated runs to formally characterize the stability and reproducibility of LLM-based ASPECTS assessment. Third, ChatGPT-5 was provided with only four representative axial NCCT slices (two ganglionic and two supraganglionic levels) rather than the entire scan volume. Although these standardized planes generally capture the bulk of MCA territory ischemia and allow consistent comparison across cases, restricting the input to four 2D images may reduce sensitivity for very small or subtle infarcts located outside these slices and may partly explain the slightly higher ASPECTS values assigned by ChatGPT-5 compared with the neuroradiology consensus. Future multimodal LLM workflows that can directly ingest and interpret the full NCCT volume (e.g., slice-wise or 3D volumetric analysis of the entire DICOM series) may enable more comprehensive lesion detection and more accurate regional scoring. Fourth, the model operated without access to clinical context—information that human readers routinely incorporate into image interpretation. Fifth, the binary ASPECTS threshold (ASPECTS < 7 and ≥7) may have reduced prognostic sensitivity in this cohort, as few patients had low scores. Future studies should assess alternative cutoffs (e.g., ≤6) and explore continuous relationships between ASPECTS and outcomes. Finally, ChatGPT-5, like other LLMs, operates as a closed-source ‘black box’ system and remains susceptible to variability and potential interpretive errors. Occasional errors or inconsistencies cannot be completely ruled out, especially as future model updates may alter its behavior. Incorporating interpretability methods, such as visual attention mapping, may help clarify model decision pathways. These approaches could also improve transparency and user trust in LLM-based imaging systems.

## 5. Conclusions

ChatGPT-5 demonstrated high reliability and strong prognostic validity in automated ASPECTS assessment on NCCT for AIS. Its near-human performance achieved without task-specific training highlights the expanding role of multimodal LLMs in quantitative image interpretation. Although these findings support the feasibility of LLM-based stroke imaging analysis, clinical implementation will require multicenter prospective validation, standardized prompting frameworks, and formal regulatory validation before integration into clinical workflow.

## Figures and Tables

**Table 1:** Baseline Clinical and Angiographic Characteristics of the Study Population.

Variable	Value
Age, years (mean ± SD)	70.2 ± 12.5
Sex, male/female, *n* (%)	101/98 (50.8/49.2)
Hypertension, *n* (%)	135 (67.8)
Diabetes mellitus, *n* (%)	68 (34.2)
Coronary artery disease, *n* (%)	63 (31.7)
Hyperlipidemia, *n* (%)	24 (12.1)
Atrial fibrillation, *n* (%)	76 (38.2)
Intravenous thrombolysis, *n* (%)	21 (10.6)
Side of stroke (right/left)	111/88 (55.8/44.2)
Occlusion site, *n* (%)	
ICA (%)	28 (14.1)
ICA + MCA M1 (%)	24 (12.1)
ICA + MCA M2 (%)	3 (1.5)
MCA M1 (%)	129 (64.8)
MCA M2 (%)	14 (7.0)
MCA M2–M3 (%)	1 (0.5)

Note: Values are presented as mean ± standard deviation (SD) or number (percentage). ICA = internal carotid artery; MCA = middle cerebral artery.

**Table 2:** Interobserver Agreement and Categorical Concordance for ASPECTS assessment.

Comparison	ICC (95% CI) *	*p*-Value	Interpretation	Cohen’s κ † (95% CI)	Agreement Level
Radiologist vs. Neurologist	0.854 (0.797–0.894)	<0.001	Excellent	0.83 (0.75–0.89)	Almost perfect
ChatGPT-5 vs. Consensus	0.845 (0.792–0.884)	<0.001	Excellent	0.79 (0.71–0.86)	Substantial
ChatGPT-5 vs. Radiologist	0.821 (0.765–0.868)	<0.001	Good	0.76 (0.67–0.84)	Substantial
ChatGPT-5 vs. Neurologist	0.807 (0.745–0.856)	<0.001	Good	0.74 (0.65–0.82)	Substantial
Overall (three raters)	0.451 (0.335–0.555)	<0.001	Poor overall multi-rater consistency	—	—

* Intraclass correlation coefficient (ICC [2,1]), two-way random-effects model, absolute agreement. † Cohen’s κ calculated for dichotomized ASPECTS (<7 vs. ≥7). Interpretation of ICC: <0.50 = poor; 0.50–0.75 = moderate; 0.75–0.90 = good; >0.90 = excellent. Interpretation of κ: <0.20 = slight; 0.21–0.40 = fair; 0.41–0.60 = moderate; 0.61–0.80 = substantial; 0.81–1.00 = almost perfect. *p*-values test the null hypothesis of no agreement (ICC = 0 or κ = 0).

**Table 3:** Multivariable Logistic Regression Analysis for Predictors of Functional Independence (mRS ≤ 2 at 3 Months).

Variable	ChatGPT-5 ASPECT (OR [95% CI])	*p*-Value	Consensus ASPECT (OR [95% CI])	*p*-Value
ASPECT score (per 1-point increase)	1.28 (1.09–1.52)	0.004	1.31 (1.11–1.54)	0.003
Age (years)	0.95 (0.92–0.98)	0.002	0.95 (0.92–0.98)	0.002
TICI 2b–3 (successful reperfusion)	2.65 (1.33–5.28)	0.006	2.67 (1.35–5.31)	0.005
Onset-to-groin time (min)	0.88 (0.74–1.04)	0.12	0.87 (0.73–1.03)	0.11
Sex (male vs. female)	1.12 (0.64–1.98)	0.70	1.10 (0.63–1.96)	0.72
Diabetes mellitus	0.93 (0.51–1.70)	0.81	0.92 (0.50–1.68)	0.82
Hypertension	0.87 (0.47–1.61)	0.65	0.85 (0.46–1.58)	0.66

CI = confidence interval; OR = odds ratio; TICI = Thrombolysis in Cerebral Infarction. Data are presented as OR (95% CI). For continuous variables, ORs correspond to a 1-unit increase (age in years, onset-to-groin time in minutes). *p*-values are derived from multivariable logistic regression models.

## Data Availability

The original contributions presented in this study are included in the article. Further inquiries can be directed to the corresponding author.

## Abbreviations

The following abbreviations are used in this manuscript:

AIS	Acute ischemic stroke
AI	Artificial intelligence
ASPECTS	Alberta Stroke Program Early CT Score
AUC	Area under the curve
DL	Deep learning
EIC	Early ischemic change
HT	Hemorrhagic transformation
ICC	Intraclass correlation coefficient
LLM	Large language model
mRS	Modified Rankin Scale
mTICI	Modified Thrombolysis in Cerebral Infarction
NCCT	Non-contrast computed tomography
OR	Odds ratio
RAG	Retrieval-augmented generation
ROC	Receiver operating characteristic
SD	Standard deviation
